# A Medico-Legal Treatise on Malpractice and Medical Evidence

**Published:** 1860-05

**Authors:** 


A Medico-Legal Treatise on Malpractice and Medical Evidence: Comprising the elements of Medical Jurisprudence. By John J. Elwell, M. D., Member of the Cleveland Bar. " A doctor who knows nothing of law, and a lawyer who knows nothing of medicine; are deficient in essential requisites of their profession."—*David Paul Brown.* New York: John S. Vorher, No. 22 Nassau Street. 1860.

We were agreeably surprised the other day by receiving the above volume of five hundred and eighty-eight pages, handsomely put up in law binding. And on opening its pages we naturally were anxious to know if the author possessed the qualifications requisite to the accomplishment of the object proposed. But we soon learned, that in addition to the high position he had attained in the profession of his choice, the advantages of a good medical education, and the experience of several years as a reputable medical practitioner, had been brought to bear in the elaboration of the present work. From a general perusal we are satisfied that the author has rendered valuable service, especially to our profession, by embodying in a concise, complete and comprehensive form, all the well established principles and known authorities, aided by his own thought and experience, upon the subjects of malpractice and medical evidence. In the discussion of the subject of the first part of the work, the author has supplied to the members of the twin professions, a disideratum that is now beginning to be felt, from the frequency, importance, and troublesome character of cases of alleged malpractice.

In the opening chapter, the author proceeds to give the general principles of law applicable to medical responsibilities "as held by the courts of England and this country, with a number of references to adjudicated cases in which medical men have been tried for mal-practice; then in a series of chapters, the difficulties peculiar to our profession, its possibilities and impossibilities; what the general practitioner can do, and what he cannot," are set forth as concisely as possible.

The subjects of amputations, fractures, and dislocations—the treatment of which has always been subject to so many embarrassments to the ordinary practitioner—are fully treated of, and "an exhibit of the present state of the science of Surgery, so that just what should be rightfully expected and required of the surgeon may be understood as far as possible, and what should excuse an imperfect result in his treatment."

We also find an interesting digest of Prof. F. H. Hamilton's able and valuable work on deformities after fractures, and we are satisfied that no one can fully realize the obligations that are due the indefatigable labors of Prof. Hamilton, without a thorough examination and study of the facts and conclusions that are so fully elaborated. The chapter on the responsibilities of Druggists, with leading cases, closes the part of the work devoted to civil malpractice.

Part second is devoted to the consideration of the leading points and subjects involved in medical evidence. The rights and duties of the medical man, while discharging the obligations of a witness, are fully and explicitly set forth in the chapters on the subjects of "Evidence in general; Circumstantial Evidence; the Testimony of Experts; Privileged Witnesses and Communications, and Medical Books as evidence."

"Also all the various medico-legal subjects that are constantly engaging the attention of the courts, and the medical witness," are fully considered. The question of Insanity is examined at considerable length, with important cases; also the subject of Poisoning by Arsenic and Strychnia in particular, with leading cases.

The subjects of Wounds, Rape, and Coroner's Inquests, make up the last part of this interesting volume. In the examination of the above work, will be found but little to criticise, but much to commend, especially as the author claims the consideration of originality in the general plan and discussion of the subjects.

